# Genetics and Therapeutic Responses to Tumor-Infiltrating Lymphocyte Therapy of Pancreatic Cancer Patient-Derived Xenograft Models

**DOI:** 10.1016/j.gastha.2022.07.006

**Published:** 2022-07-15

**Authors:** Lisa M. Nilsson, Caroline Vilhav, Joakim W. Karlsson, Johan Bourghardt Fagman, Daniel Giglio, Cecilia E. Engström, Peter Naredi, Jonas A. Nilsson

**Affiliations:** 1 Department of Surgery, Sahlgrenska Center for Cancer Research, Institute of Clinical Sciences, Sahlgrenska Academy, University of Gothenburg and Sahlgrenska University Hospital, Gothenburg, Sweden; 2 Harry Perkins Institute of Medical Research, University of Western Australia, Perth, Australia; 3 Department of Oncology, Institute of Clinical Sciences, Sahlgrenska Academy, University of Gothenburg and Sahlgrenska University Hospital, Gothenburg, Sweden

**Keywords:** Patient-Derived Xenografts, Pancreatic Cancer, Tumor-Infiltrating Lymphocytes, Transcriptomic classification

## Abstract

**Background and Aims:**

Pancreatic cancer is the seventh leading cause of cancer-related deaths worldwide. Checkpoint immunotherapy has not yet shown encouraging results in pancreatic cancer possibly because of a poor immunogenicity and/or an immune suppressive microenvironment. The aim of this study was to develop patient-derived xenograft (PDX) models, compare their genetics to the original biopsies, and assess if autologous tumor-infiltrating lymphocytes (TILs) would have antitumoral activity in pancreatic cancer.

**Methods:**

We subcutaneously transplanted tumors from 29 patients into NOG mice to generate PDX models. We established TIL cultures and injected them into PDX mice. We analyzed histology and genetics of biopsies and PDX tumors.

**Results:**

Tumor growths were confirmed in 11 of 29 transplantations. The PDX tumors histologically resembled their original biopsies, but because stromal cells in the PDX model tumors were from mouse, their gene expression differed from the original biopsies. Immune checkpoint ligands other than programmed death ligand-1 (PD-L1) were expressed in pancreatic cancers, but PD-L1 was rarely expressed. When it was expressed, it correlated with tumor take in PDX models. One of the 3 tumors that expressed PD-L1 was an adenosquamous cancer, and another had a mismatch repair deficiency. TILs were expanded from 6 tumors and were injected into NOG or human interleukin-2 transgenic-NOG mice carrying PDX tumors. Regression of tumors could be verified in human interleukin-2 transgenic-NOG mice in 3 of the 6 PDX models treated with autologous TILs, including the adenosquamous PDX model.

**Conclusion:**

PDX models of pancreatic cancer can be used to learn more about tumor characteristics and biomarkers and to evaluate responses to adoptive cell therapy and combination therapies. The major benefit of the model is that modifications of T cells can be tested in an autologous humanized mouse model to gain preclinical data to support the initiation of a clinical trial.

See editorial on page 1122.

## Introduction

Pancreatic cancer is increasing in incidence and is now the seventh leading cause of cancer-related deaths worldwide.^1^ However, in more developed countries, it is more frequent, and in the United States, pancreatic cancer is the third most common cause of cancer-related deaths.^2^ The treatment options and possibility for cure are increasing in many types of cancer, but for pancreatic cancer, only small advances have been made, and the 5-year survival rate is still only 2%–9% in the overall population.^3^

Cancer cells can elicit an immune attack, so in order for a tumor to grow, various immune evasion mechanisms need to operate. The protein programmed death ligand-1 (PD-L1) is expressed on the surface of many tissues, including endothelial and epithelial cells. Activated T cells express PD-1 receptors, and when binding to PD-L1, they become inactivated, or exhausted, preventing them from destroying normal tissue. Tumor cells are normally treated as invaders in the body, being attacked and destroyed by T cells. In certain circumstances, tumor cells can achieve the capacity to overexpress PD-L1 on their surface and inactivate the T cells.^4^^,^^5^ Expression of PD-L1 in tumors is consequently an indirect sign of T-cell activity.

Immune evasion can be targeted in cancer therapy, which is called immunotherapy. PD-L1/PD-1 on the tumor cells or T cells can be blocked with immune checkpoint inhibitors. These inhibitors have dramatically altered the treatment landscape of malignant melanoma, kidney cancer, leukemia, and many other malignancies but have not yet shown positive results in pancreatic cancer.^6^^,^^7^ Research is ongoing to understand the underlying mechanisms of anti-PD-1/PD-L1 monotherapy failure. Possibly, it might be because of a unique metabolism or immunosuppressive tumor microenvironment, which antagonizes the immune cells.^8^, ^9^, ^10^ However, immunotherapy resistance mechanisms are complex, and more studies are necessary to understand processes ongoing in the pancreatic tumors. Combination strategies for overcoming resistance to immune checkpoint inhibitor are being investigated.^11^^,^^12^

Patient-derived xenografts (PDXes) are mouse models established by implantation of human cancer tissue into immunodeficient NOD/SCID interleukin-2 (IL-2) receptor knockout mice (NOG/NSG mice). The tumors in these models preserve the original histology and genetic heterogeneity during serial transplantation.^13^ Because of their similarities to human cancer, PDXes are often preferred over cell line–derived xenografts in drug efficacy and biomarker studies.^14^ Moreover, PDXes can be used to guide therapy in diagnoses producing rapidly developing models.^15^, ^16^, ^17^ Initially though, PDX models were not useful in immunotherapy research because of the immunodeficient nature of the mice. Recently, new models have been developed with varying degrees of immune humanization.^18^ As a result of this, PDX models have also, in some cases, been shown to also accurately predict immunotherapy responses in patients.^19^

Adoptive cell therapy (ACT) is an immunotherapy where T cells, for example, tumor-infiltrating lymphocytes (TILs), are grown out from a patient’s tumor or blood, expanded in vitro in the presence of human IL-2, and later infused back into the same patient.^20^ This therapy can cause durable responses in patients, although it is labor intensive and requires specialized infrastructure. Thus far, most trials have been conducted in melanoma, renal cell carcinoma, and ovarian cancer, but TILs are present in pancreatic cancer and can be expanded ex vivo.^21^

To learn more about the potential of ACT with TILs in human cancer, we recently developed a new model called PDXv2.^22^ By transplanting tumors into NOG mice transgenic for human IL-2 (hIL2-NOG), we were able to promote the survival and proliferation of injected TILs over a long time, without need to inject exogenous IL-2. This causes responses to TIL therapy that mimic the outcome in the clinical ACT trial from which the tumors and TILs were obtained.^22^ This indicates that PDXv2 is a model in which tumor-killing activities of TILs and how they synergize with other treatments^19^^,^^22^, ^23^, ^24^ can be studied. Here, we xenotransplant human pancreatic tumor biopsies into NOG and hIL2-NOG mice and generate TILs from the same biopsies to investigate if ex vivo expanded TILs from pancreatic cancer can cause responses in PDXv2 mice.

## Materials and Methods

### Patients and Ethics

Thirty patients with suspected pancreatic cancer, scheduled for surgery intended to cure at Sahlgrenska University Hospital, Gothenburg, Sweden, were included in the study. One patient had to be excluded because of logistical problems during the operation. Patient characteristics of the residual 29 patients, considering age, sex, pathological examination, TNM stage, tumor differentiation, time of recurrence, and death, are presented in Tables 1 and 2. A prospective chart review was undertaken. Institutional review board approval was obtained for both PDX generation and review of clinical data in medical records (Gothenburg ethics committee approvals #091-13 and #057-18).

**Table 1:** Patient Characteristics for Tumors in Successful PDX Models

Patient	Age^a^	Sex	Recurrence^b^	Death^c^	Tumor type	Differentiation	pTNM^d^
PaC05	67	M	786	1100	PDAC	Medium	T3N1
PaC06	47	M	100	205	PDAC	Low	T3N1
PaC07	70	F	147	430	PDAC	Medium	T3N1
PaC10	68	M	348	409	ASCAP	N/a	T3N1
PaC16	61	M	10	584	PDAC	Medium	T2N0
PaC17	61	F	152	298	PDAC	Medium	T3N1
PaC24	69	M	212	331	PDAC	Medium	T3N0
PaC25	76	M	163	206	Ampullary adenocarcinoma	Medium	T3N1
PaC26	61	F			PDAC	Medium	T3N0
PaC29	62	F			PDAC	Medium	T3N1
PaC30	61	M			Cholangiocarcinoma	Low	T3N1

F, female; M, male.

a Age at the time of surgery.

b Time in days from surgery to recurrence.

c Time in days from surgery to death.

d Seventh edition.

**Table 2:** Patient Characteristics for Tumors in Nonsuccessful PDX Models

Patient	Age^a^	Sex	Recurrence^b^	Death^c^	Tumor type	Differentiation	pTNM^d^
PaC01	77	F		654	PDAC	Medium	T3N1M0
PaC02	70	F		106	PDAC	Medium	T3N1M0
PaC03	74	M	63	82	PDAC	Low	T3N1M0
PaC04	70	F	756	1049	PDAC	High	T3N1M0
PaC09	71	M	778		PDAC	Low	T2N1M0
PaC11	78	M			Ampullary adenocarcinoma	Low	T3N0M0
PaC12	60	F			PDAC	High	T1N1M0
PaC13	60	F	387	601	PDAC	High	T3N1M0
PaC14	74	F	427	995	PDAC	Low	T1N0M0
PaC15	59	F			Chronic pancreatitis	-	-
PaC18	79	M			PDAC	Medium	T2N1M0
PaC19	78	F	333	713	PDAC	High	T1N1M0
PaC20	62	M		520	PDAC	Medium	T1N1M0
PaC21	74	F	N/a	244	Lever metastasis PDAC	-	T3NXM1
PaC22	66	F			PDAC	Medium	T2N1M0
PaC23	63	M			IPMN high degree dysplasia	-	TisN0M0
PaC27	61	F	59	172	Ampullary adenocarcinoma	Low	T3N1M0
PaC28	75	M		786	Cholangio-carcinoma	Low	T3N0M0

F, female; M, male.

a Age at the time of surgery.

b Time in days from surgery to recurrence.

c Time in days from surgery to death.

d Seventh edition.

### Preprocessing of RNA Sequencing Data

Reads were aligned to the hg19 reference human genome, excluding alternative haplotype regions, with HISAT v. 0.1.6-beta^25^ (parameters: --no-mixed --no-discordant --no-unal --known-splicesite-infile, using splice junctions defined in the GENCODE v. 19 human genome annotation). PDX samples were also aligned to the GRCm38 reference genome separately. Alignments were converted to BAM format, sorted, and indexed using samtools v. 0.1.19.^26^ Human reads were distinguished from mouse reads using Disambiguate v. 1.0 (parameter: “-a hisat2”). Gene-level read counts were quantified using HTSeq (v. 0.11.2),^27^ with the script “htseq-count” and the parameters “-m intersection-strict -s no.” RPKM normalized values were calculated, taking into account the maximal mature transcript length of each gene.

### Transcriptomic Classification

The Cancer Genome Atlas (TCGA) data were downloaded and processed as described previously.^28^ Pairwise Spearman correlation coefficients were calculated between our sample and each TCGA sample, with respect to all coding genes. Classification was performed using a k-nearest neighbor approach based on these correlation coefficients, using k = 6, as previously found to be optimal based on leave-one-out cross-validation on the TCGA cohort.^28^ With this approach, any ties are broken by taking the majority vote after removing the worst correlated sample. Further subtype analysis was carried out using metadata collected from a previous study on the TCGA pancreatic adenocarcinoma (PAAD) cohort.^29^ A similar approach as mentioned earlier was used to classify our samples relative to subgroups defined based on this metadata. First, leave-one-out cross-validation was performed using each TCGA-PAAD sample to determine the accuracy in predicting each metadata category. The value of *k* that achieved the highest accuracy across all assessed categories (k = 15) was chosen for use with our samples. Then, for each of our samples, a ranked list of TCGA-PAAD samples was obtained based on Spearman correlation coefficients, after which the majority vote among the top k samples was selected as the classification. This was done separately for patient biopsies and PDX samples to compare differences in associations to known subgroups.

### Immune Cell Deconvolution

To determine cell types contributing to the composition of bulk RNA-seq samples, RPKM normalized gene expression values were used as input to the function deconvolute from the R package immunedeconv (v. 2.0.3), which contains wrapper functions to run a number of different cell-type deconvolution methods. The parameter “method = ‘epic’” was used to run EPIC (v. 1.1.5).^30^

### Preprocessing of Exome Sequencing Data

Exome sequencing reads were aligned to the 1000 genomes version of the hg19 human reference genome (v. 37) with bwa ^31^ using the arguments “mem -t 10 -M -R.” PDX samples were also separately aligned to the GRCm38 reference genome. Alignments corresponding to multiple sequencing runs of the same sample were merged using the samtools “merge” command (v. 1.9). For PDX samples, human reads were distinguished from mouse reads using Disambiguate (parameter: -a bwa). Duplicate reads were marked with MarkDuplicates (GATK v. 4.1.3.0) ^32^ using default parameters. Base quality score recalibration was performed with BaseRecalibrator and ApplyBQSR (GATK) in 2 passes using the same reference genome as well as lists of known polymorphisms from the GATK resource bundle (files “dbsnp_138.b37.vcf,” “1000G_phase1.indels.b37.vcf,” and “Mills_and_1000G_gold_standard.indels.b37.vcf”).

### Mutation Calling

Variant calling for exome sequencing alignments was performed with Mutect 2^33^ (GATK v. 4.1.3.0) using the parameters “--genotype-germline-sites true --genotype-pon-sites true --af-of-alleles-not-in-resource 0.0000025 --disable-read-filter MateOnSameContigOrNoMappedMateReadFilter.” The GnomAD^34^ population variant database was provided as a germline resource, together with the same reference genome as mentioned earlier. The analysis was restricted to exome target regions corresponding to Agilent SureSelect Clinical Research Exome v2 or Twist Exome depending on sequencing batch. In addition, a panel of normals was supplied as input built from all available normal samples in the study. This panel was built by first running Mutect 2 in tumor-only mode on each normal with the parameter “--disable-read-filter MateOnSameContigOrNoMappedMateReadFilter” and then running CreateSomaticPanelOfNormals (GATK) on the resulting files. Variant quality labels were assigned using FilterMutectCalls (GATK) using the same reference genome as previously. These variants were then annotated using the script vcf2maf.pl (https://github.com/mskcc/vcf2maf), which relies on VEP, using the v. 98 build of the VEP reference database for the GRCh37 genome. Variants were further filtered using custom scripts to remove genes with >0.001 frequency in GnomAD, ExAC, genes with dbSNP identifiers, unless any of these variants were whitelisted. Variants were whitelisted if they either were listed as oncogenes Cancer Gene Census genes and the exact mutation was listed in COSMIC; if they were listed as tumor suppressors in Cancer Gene Census. The resulting list was further filtered to remove variants that only occurred in more than one PDX sample but not in any patient biopsy.

Sanger sequencing of *KRAS* was made in the 11 established PDX models (Table 3). Next-generation sequencing, gene signature, and mutational analysis were performed on DNA and RNA prepared from patients’ blood or xenograft biopsies.

### Copy Number Analysis

Copy number segmentation was performed with CNVkit (v. 0.9.6). A reference was prepared with the commands access and autobin using default parameters. Then the coverage command was used, supplying matching tumor and normal files, exome target regions based on the kit used (Agilent SureSelect Clinical Research Exome v2 or Twist Exome), the 1000 Genomes version of the hg19 human reference genome (v37), and a list of problematic regions to exclude (http://hgdownload.cse.ucsc.edu/goldenpath/hg19/encodeDCC/wgEncodeMapability/wgEncodeDukeMapabilityRegionsExcludable.bed.gz). This was followed by using the command fix to correct for coverage biases and GC content, segmentation with the segment command. The resulting output was converted to SEG-formatted files using the commands “cnvkit.py segmetrics” (parameters: “--ci -a 0.05”) followed by “cnvkit.py call” (parameters: “--center “median” --purity 1 --filter ci”) and “cnvkit.py export seg.”

### Generation of PDX Models and Adoptive Cell Transfer

The experiments were performed in accordance with EU directive 2010/63 (regional animal ethics approval #36-2014 and #1183-2018). Each patient material was implanted subcutaneously in 2 NOG mice (nonobese severe combined immune-deficient IL-2 chain receptor γ knockout mice, Taconic, Denmark). The growth of tumors was followed with caliper measurements weekly, and tumor volumes were calculated according to the formula width × width × length/2. For serial transplantation, the tumor was harvested and transferred into new recipient animals. Some animals were also transplanted orthotopically. Mice were anesthetized, and a small incision was made, enabling injection of chopped subcutaneous PDX tumor into the pancreas of the mouse.

**Figure 1:**
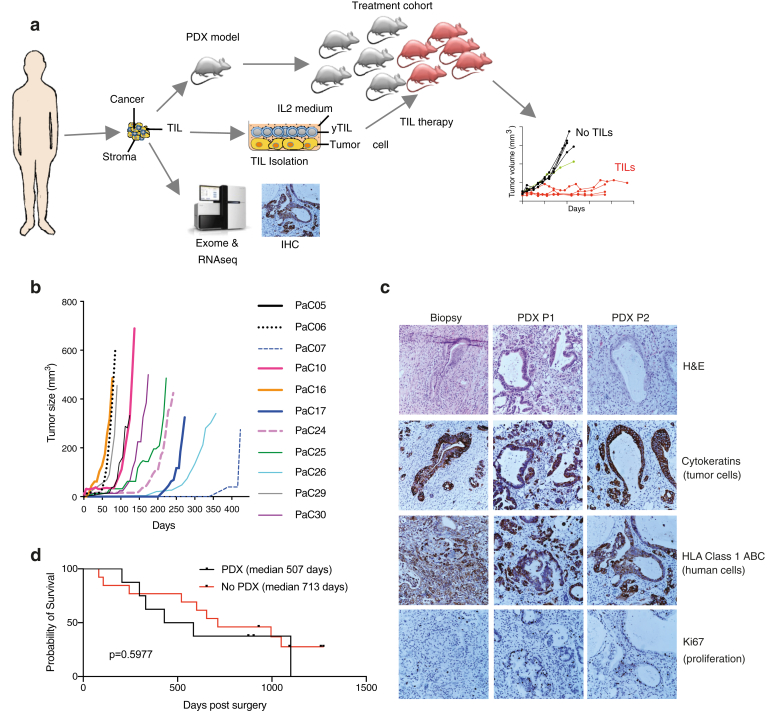
Establishment of pancreatic cancer PDX models for cellular immunotherapy studies. (A) Schematic presentation of the project. Tumors are used for generation of PDX models, DNA/RNA for sequencing, immunohistochemistry (IHC), and TILs for later immunotherapy. TIL therapy is monitored using caliper measurements of tumors growing in PDX mice. (B) Tumor growth curves of tumors from 11 patients. (C) Representative IHC of pancreatic cancer in patients and in 2 passages in mice. Staining was performed with H&E or antibodies against human cytokeratins, HLA-ABC, and Ki67. Another patient sample is seen in Figure A1. (D) Survival of patients with PDAC in the cohort divided into whether tumors formed (n = 8) or not (n = 13). H&E, hematoxylin and eosin; IL2, interleukin 2.

TIL expansion was performed on fresh biopsies where small tumor pieces were cultured for 3–4 weeks in RPMI medium containing 10% human serum and 6000 U/mL IL-2. The yTILs generated were further expanded in a rapid expansion protocol (REP) in the presence of CD3 antibody, irradiated feeders, human serum, and IL-2. After 14 days, the REP-TILs were counted and cryopreserved for further experiments. To generate PDXv2, a second mouse model, hIL2-NOG, was used to test the activity of the expanded TILs on their autologous tumor with ACT.^22^ Each PDX was implanted on 5 NOGs and 5 hIL-2-NOGs. When the tumors reached 80–100 mm^3^ in volume, a group of hIL-2-NOG was infused with 20x10E6 REP-TILs via tail-vein injection (Figure 1A). hIL2-NOG mice not receiving TIL infusion were followed as no TIL controls.

### Immunohistochemistry

The tumor tissues were fixed in formalin, sectioned into 4 μm slides, and stained with standard hematoxylin and eosin, human cytokeratins, human leukocyte antigen (HLA)1 A, B, C, PD-L1, and Ki67.

To further characterize the xenograft tissue from mice injected with TILs, we stained PDXv2 tumors with antibodies directed against human CD3, CD8, and CD137.

### Statistical Analyses

Tumor growth curves were plotted in GraphPad Prism 9, and statistical analyses were performed using the multiple t test function.

## Results

### Clinical and Genetic Characteristics of Pancreatic Cancer PDX Models

Thirty consecutive patients scheduled for pancreaticoduodenectomy due to suspected pancreatic cancer underwent surgery at Sahlgrenska University Hospital. After the specimens were removed perioperatively, the biopsies were sent to pathology, and small pieces were saved for research (Figure 1A). The pathological examination (Table 1) determined that the vast majority were pancreatic ductal adenocarcinoma (PDAC; n = 21), whereas the rest were ampullary adenocarcinoma (n = 3), cholangiocarcinoma (n = 2), intraductal papillary mucinous neoplasm (n = 1), adenosquamous carcinoma of the pancreas (ASCP; n = 1), and chronic pancreatitis (n = 1). Subcutaneous transplantations were performed on 2 immunocompromised NOG mice per patient specimen, and several small pieces were dispersed in cell culture plates for generation of TILs in the presence of human IL-2, whereas other pieces were subjected to DNA and RNA preparation. Twenty-nine of the biopsies were transplanted, and 11 exhibited subcutaneous growth (Figure 1B), sometimes in both mice transplanted with the patient biopsy. To validate the accuracy of the PDX models, paraffin-embedded tumors were stained with hematoxylin and eosin, human cytokeratins, HLA class 1, and the proliferation marker Ki67 in every serial transplantation step. The staining verified the consistency with the original patient tumor biopsies with respect to tumor cells, persistence of human cells, high amount of stroma, and proliferation pattern (eg, see Figure 1C). Histology did not overtly differ between subcutaneous or orthotopic transplantations (Figure A1). At variance with melanoma,^35^ where PDX model take rate and growth rate correlate with survival, there was no statistical difference in survival between patients with PDAC whose biopsies grew in mice or not (Figure 1D).

**Figure 2:**
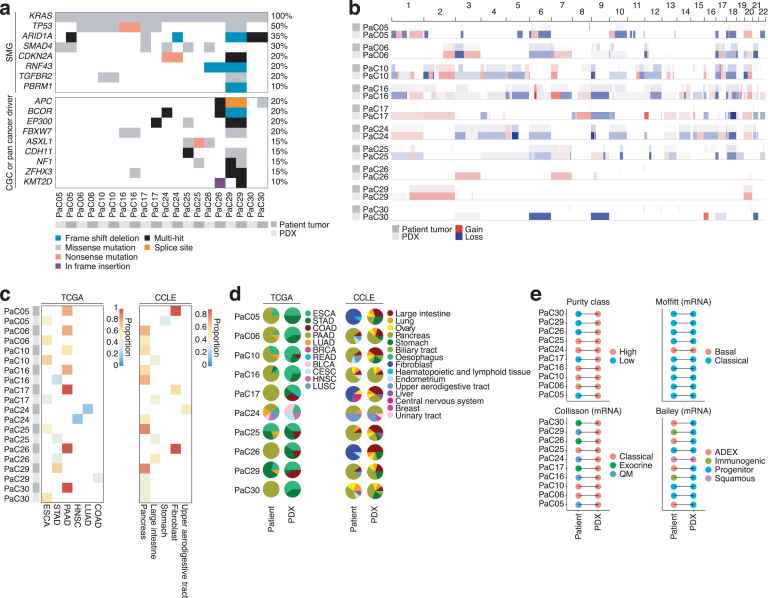
Somatic genomic alterations and transcriptomic classification. (A) Mutations in genes that have either previously found to be significantly mutated in pancreatic ductal adenocarcinoma, which are listed in the COSMIC Cancer Gene Census (CGC) or which have been described as pan-cancer drivers.^29^ For genes in the latter 2 categories, only those mutated in at least 2 independent samples are shown. (B) Copy number alterations, shown as color-coded in proportion to log_2_ ratios of tumor relative to normal. Red indicates gains and, blue indicates losses. (C) Predicted cancer or cell types for each sample based on *k*-nearest neighbor classification (*k*-NN; *k* = 6^28^) on the basis of Spearman correlation coefficients calculated with respect to all coding genes, using either a pan-cancer data set from TCGA as reference or the Cancer Cell Line Encyclopedia (CCLE, Table A1). The colors indicate the proportion among the top 10 most strongly correlated samples that are the same cancer type as the final prediction, as a measure of agreement. (D) As in (C), but showing the proportions of each tumor or cell type that occurred among the top 10 samples in the ranked list used for classification of a given tumor. (E) Classification of pancreatic cancer subtypes based on metadata from a previous TCGA study, using *k*-NN classification with *k* = 15 (found to be optimal in leave-one-out cross-validation on the TCGA-PAAD cohort; Figure A3A).

The PDX tissues and matching patient biopsies were subjected to exome and RNA sequencing. Overall mutational load was low for all except one sample, PaC29, which had a mutational signature associated with defective mismatch repair, as well as a germline mutation in and low expression of the mismatch repair protein MSH6. As expected, *KRAS* mutations^36^ was observed in all tumors, and this was confirmed by Sanger sequencing (Figure 2A and Table 3). The second most mutated gene was *TP53*, but also the tumor suppressor *SMAD4* had mutations, which interestingly was more manifest in PDX tissue than in the original patient biopsy. This could be due to a subclone that grew better in mice or because the amount of stroma in the patient biopsy interfered with mutation calling. Indeed, copy number assessment (Figure 2B) consistently produced more distinct copy number variations in the PDX samples.

**Table 3:** Genomic and Immunological Characteristics of Xenograft Tissue

Sample	KRAS mutation (Sanger^a^ and NGS)	yTILs^b^	PDXv2^c^
PaC05	G12V (G:T)^a^	Yes	Responder
PaC06	Q61H	Yes	Nonresponder
PaC07	G12V (G:T)^a^	No	N.A.
PaC10	G12D (G:A)^a^	Yes	Responder
PaC16	G12D (G:A)^a^	Yes	Responder
PaC17	G12V (G:T)^a^	Yes	Nonresponder
PaC24	G12D (G:A)^a^	No	N.A.
PaC25	G12D (G:A)^a^	Yes	Nonresponder
PaC26	G12D (G:A)^a^	No	N.A.
PaC29	G12A (G:C)^a^	No	N.A.
PaC30	G12D (G:A)^a^	No	N.A.

N.A., not analyzed.

a Confirmation by Sanger sequencing.

b Young tumor-infiltrating lymphocytes isolated from primary patient tumor samples.

c Assessment of tumor regression of PDX, tumors in response to infusion with autologous y-TILs.

Transcriptomic classification is a powerful bioinformatic tool that can be used to assess diagnosis or characterize tumors. When comparing the RNA-seq data of the pancreatic cancer PDX and patient biopsies to the tumor RNA-seq data of TCGA using a previously published bioinformatic workflow,^28^ not a single of the PDX models is predicted to be pancreatic adenocarcinoma (PAAD in the TCGA). Rather, the PDX transcriptomes correlated more with those of other gastrointestinal tumors in TCGA such as esophageal or stomach cancer (Figure 2C and D). However, when comparing to the Cancer Cell Line Encyclopedia (CCLE), also PDX samples are predicted to resemble pancreas cancer. This suggests that the stroma compartment in PDX models is lost, and this affects overall gene expression. Indeed, 3 patient biopsies were most similar to fibroblast cell lines of the CCLE (Figure 2C and D), demonstrating the important contribution of stromal cells of mesenchymal origin in the pancreatic cancer tumors. Subgroup analysis of the samples using metadata from TCGA showed that only the Moffit classification scheme had any accordance between PDX model and patient biopsy (Figure 2E and Figure A2). Collectively, the analyses show that PDX models can be valuable to identify copy number variations and clonal mutations of relevance (eg, SMAD4) and that the transcriptome is coming only from the pancreatic cancer cells and not from stroma. Hence, the accurate histology of PDX models (Figure 1C) is therefore due to that mouse stroma cells are recruited by the pancreatic cancer cells to build up the tumor, which we verified using mouse vimentin staining and lack of human HLA-A, B, C expression (Figure A1 and data not shown).

### Immunology and Immunotherapy of Pancreatic Cancer PDX Models

**Figure 3:**
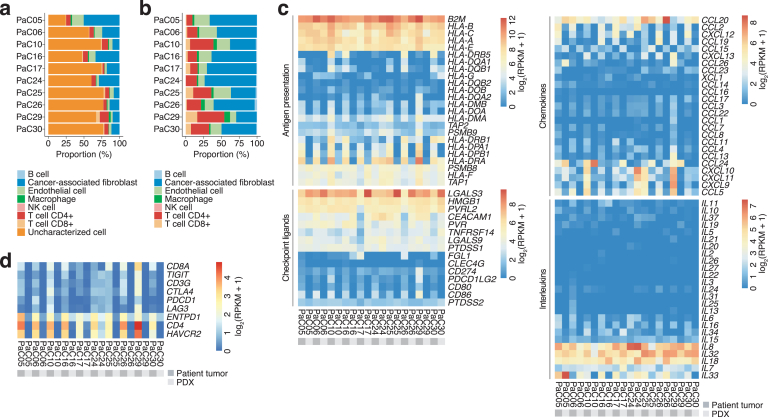
RNA-based immune profiling. (A) Inference of the proportions of immune cell types, as well as cancer-associated fibroblasts and endothelial cells present in samples, based on deconvolution of bulk RNA-seq data of patient biopsies using EPIC.^30^ (B) As in (A), but excluding uncharacterized cells (likely to be cancer cells). (C) Expression levels of genes involved in interactions with immune cells. (D) Expression levels of genes encoding immune checkpoint proteins. A list of genes describing HLA genes and immune checkpoint ligands are present in Table A2.

The RNA-seq data from the biopsies enabled analyses into the transcriptional expression of immune-related factors in pancreatic cancer. We used EPIC as cell-type deconvolution tools to show that although the majority of reads appeared to come from “uncharacterized cells,” most likely cancer cells, and “cancer-associated fibroblasts,” ie, stroma cells, most samples did have a fraction of immune cells (Figure 3A). Excluding uncharacterized reads generated a breakdown and confirmation of the strong stromal compartment in the biopsies. Three samples were predicted to have more T-cell infiltration: PaC10, PaC25, and PaC29 (Figure 3B). B cell infiltration was seen to a small degree in PaC10, PaC25, and PaC26, which also had somewhat more prominent expression of HLA Class 2 genes (Figure 3C). Class 1 and antigen presentation genes (eg, *TAP1* or *PSMB8*) were expressed in both patient biopsies and PDX tumors, whereas Class 2 genes and immune checkpoint proteins were only expressed in patient biopsies (Figure 3C and D). This confirms studies in melanoma PDX models that show that immune cells do not survive in PDX models.^22^

We also performed cell-type deconvolution using the commonly used tool CIBERSORT. This tool gives somewhat different results. In particular, CIBERSORT estimates much higher macrophage fractions than EPIC for these samples, which is not consistent with immunohistochemistry. Interestingly, these fractions seemed to be on par with the fractions of cancer associated fibroblasts (CAFs) predicted by EPIC (Figure A3A and B). We investigated the expression of different cell type markers in pancreas^37^ to assess which of these predictions is the most reasonable. Genes encoding markers of fibroblasts and mesenchymal cells are much more highly expressed than markers of macrophages or monocytes, supporting that the predictions of EPIC are more likely to be accurate for these samples (Figure A3C and D). This further suggests that CIBERSORT is erroneously classifying CAFs and/or mesenchymal cells as macrophages. We further investigated this by checking the correlation between fibroblast markers and different cell type proportions predicted by the 2 tools. As expected, the average of these markers (“Fibroblast score” in Figure A3E) significantly positively correlates with predicted CAF proportions for EPIC (Spearman correlation coefficient: 0.77, *P* < .01), but not with macrophage proportions (0.58, *P* < .09). For CIBERSORT, on the other hand, the fibroblast score correlates significantly positively with predicted M0 macrophage proportions (0.7, *P* < .03), which is also the most abundantly reported macrophage subset with this tool (Figure A3F).

Expression of genes encoding immune checkpoint ligands, chemokines, and ILs were also analyzed, and some of these were exclusively found in patient biopsies and not PDX models, whereas, for example, ligands for the immune checkpoints TIM3, LAG3, and TIGIT were expressed in both (Figure 3C). PD-L1 and PD-L2, the ligands for PD-1, were predominantly expressed in PaC29 and to a lesser extent in PaC10, but only in the patient biopsies. This was also verified by immunohistochemistry (Figure A4), where the 2 tumors showed strong PD-L1 expression and 1 tumor showed moderate expression. Interestingly, the biopsies expressing PD-L1 also established as PDX models, suggesting that PD-L1 positivity in the patient biopsy correlates with “take rate” in mice (*P* = .04 in a Fisher’s exact test).

**Figure 4:**
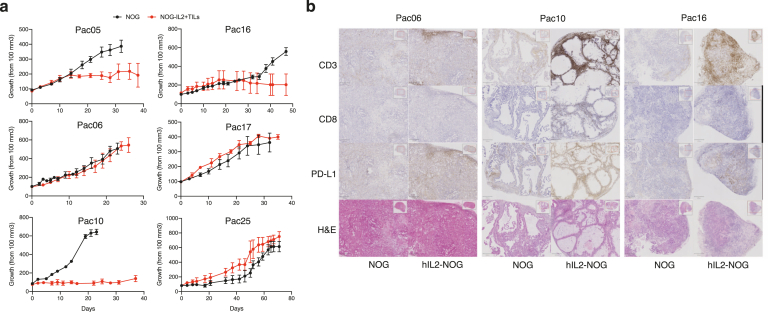
Pancreatic cancer PDX tumors (n = 3–5) growing in NOG mice or in hIL2-NOG mice and treated with autologous TILs. (A) Cumulative tumor growth curves from caliper measurements. Individual growth curves and controls where tumors are grown in hIL2-NOG without TILs are shown in Figure A4. (B) Immunohistochemistry analysis of TIL infiltration (CD3 and CD8) and expression of the immune checkpoint ligand PD-L1. Shown are a representative image of minimum 3 mice per PDX model. Higher magnification images of representative sections of all 6 PDX models are in Figures A6–A11. H&E, hematoxylin and eosin;

To assess if TILs in pancreatic cancer could be used for therapy, TILs were expanded upon the arrival of the biopsy to the laboratory. There were only 6 samples that generated both TIL cultures as well as PDX models, albeit in the case of TILs, it was not always due to that there were no TILs but because of the high number of microorganisms in some of the samples. Nevertheless, we serially transplanted the 6 PDX models into both NOG and hIL2-NOG mice. When the tumors were subcutaneously growing and reached the size of 80–100 mm^3^, the hIL2-NOG mice received a TIL infusion. NOG and hIL2-NOG mice that instead got injections with phosphate-buffered saline were used as negative controls. TIL therapy had clinical benefits in 3 of 6 PDXv2 models; PaC05, PaC10, and PaC16 (Figure 4A and Figure A5). No effect on the tumor size could be seen in the hIL2-NOG mice not receiving TILs, indicating that this is not an effect of IL-2. No tumor regression could be seen in the remaining 3 PDXv2 models, indicating that the autologous TILs did not have any effect. To investigate if the reduced tumor growth in some of the models was associated with T-cell recognition of the tumor, we investigated PD-L1 expression in the PaC06, 10, and 16 tumor models. PD-L1 is upregulated on tumor cells following exposure to interferon gamma produced by T lymphocytes^38^ and can thus provide some insights into general immune activation in the tumor environment. The PaC10 biopsy expressed PD-L1 (Figure A4), but as expected from the low RNA expression in the PDX model (Figure 3C), PD-L1 expression was low in the PaC10 model without TILs. However, after TIL injection, the tumor became very strongly positive for PD-L1 as assessed by immunohistochemistry (Figure 4B), suggesting that the TILs had been activated to secrete interferon gamma. Indeed, this induction was also seen in 2 other models, PaC05 and PaC16, that also had stunted growth by TILs, but not in the nonresponding models (Figures A6–A11). We were also able to detect weak expression of CD137 (4-1BB), a marker of activation, in TILs injected into subcutaneous and orthotopic PDX mice carrying PaC10 and PaC16, in a time-dependent manner (Figures A12 and A13).

## Discussion

The aim of the study was to create PDX models to enable research on pancreatic tumor characteristics and tumor responses to immunotherapies. Similarity between the PDXes and corresponding tumors in the patients could be verified both by immunohistochemistry and *KRAS* mutation analysis. Significant differences with regard to gene expression were also observed because PDX models have mouse and not human stromal cells. As the mouse reads are filtered out during the preprocessing, the RNA-seq data of the PDX tumors corresponds to the tumor cells only, and not tumor plus stroma as in patient biopsies. We show here, for the first time, the transcriptomic classifications of PDX models and the utility of using both the TCGA and CCLE databases for understanding the composition and gene expression profiles of pancreatic tumors.

We also compared 2 different cell-type deconvolution tools and found that EPIC was more suitable than CIBERSORT for pancreatic cancer bulk RNA-seq data. We found that data using CIBERSORT should be interpreted with caution because a misclassification of CAFs and/or mesenchymal cells cause the macrophage proportions to be overestimated. However, if the distinction between macrophages and CAFs (by considering them both as the same category) is ignored, and only cell types predicted by both are considered, then the shared cell types predicted are similar. The only exception is that CIBERSORT finds a slightly higher B cell content for some samples. Whether this would be in favor of using CIBERSORT in RNA-seq data from, for example, blood, remains to be determined. Indeed, no cell-type deconvolution method is perfect, but we can confirm the high performance of EPIC as described in a recent in-depth comparison and systematic benchmark study.^39^

Only one of the PDAC patients had high PD-L1 expression, indicative of a tumor type with generally low tumor mutational burden. PD-L1 expression in PDAC varies a lot between studies, from only 5% to 15%^40^ up to 60%–90%^41^ with different detection methods.^42^ One study described that high expression of PD-L1 associates with worse prognosis.^43^ Whether PDAC patients with high PD-L1 are more sensitive to immunosuppressive treatment and more likely benefit from anti-PD-1/PD-L1 antibodies is not fully known.^44^ The PD-L1 expression alone is not sufficient to predict patient response to immunotherapy and all patients whose tumors express membranous PD-L1 do not respond to anti-PD-1 or anti-PD-L1 therapy.^42^ The overall knowledge on the role of this pathway and its regulatory mechanisms in PDAC is still limited,^40^^,^^42^ and more studies are essential to understand the immunological processes of pancreatic cancer.

ASCP is a rare subtype of pancreatic cancer and accounts for 1%–4% of exocrine pancreatic malignancies. The prognosis of ASCP is as bad as PDAC or even worse,^45^^,^^46^ with a median overall total survival duration of 4 months and a median overall survival rate of patients with resected ASCP of 12 months.^47^ We observed that the PaC10 ASCP tumor biopsy exhibited PD-L1 expression, which confirms a recent study.^48^ The PaC10 PDX model showed regression of the tumor tissue after TIL injection. It is tempting to speculate that these patients, even if rare, might potentially benefit from PD-1 checkpoint inhibitor or TIL immunotherapy, which we are actively investigating further.

Recent studies demonstrate that the pancreatic cancer tumor microenvironment contains immune suppressive cells such as T regulatory cells and myeloid-derived suppressor cells.^49^, ^50^, ^51^ Single-cell analyses show that the likely consequence is that CD8+ cytotoxic and CD4+ helper T cells show signs of exhaustion, dysfunction, and senescence.^52^^,^^53^ High expression of many immune checkpoints such as TIGIT, PD-1, and LAG3 is present in CD8+ TILs, but only TIGIT differed significantly in expression compared with CD8+ cells from the adjacent tissue. We can confirm the expression of ligands for TIGIT, TIM3, and LAG3 in some of the biopsies^52^ and in PDX tumors. We therefore believe that our PDX models may be well suited to use as research models for drug discovery experiments to look for inhibitors of these pathways.

Despite the TILs in pancreatic cancer being exhausted, we could expand TILs from many biopsies, as has been shown by others.^21^^,^^54^ The most common reason for failure was microbial infection of the TIL culture, which could be improved by better aseptic handling of the samples by the surgeon. In 3 of 6 PDX models, the TILs that were expanded from pancreatic cancer tumors recognized the tumors, as assessed by PD-L1 and CD137 staining, which are known to be induced by interferon gamma when T cells recognize tumors, for example, in PDX models.^22^ Moreover, the TILs had the ability to suppress tumor growth in the PDX models grown in hIL2-NOG mice, indicating that there are T cells within these tumors that can be expanded and that recognize tumor cells and even control tumor growth when working under optimal conditions. Recently, Offringa et al showed that loss of tumor-dominant T-cell clones and overgrowth by newly emerging T-cell clones, which are barely detectable in the tumor, occurred during TIL expansion in vitro.^54^ By using single-cell T cell receptor and gene expression sequencing, they showed an association between poor proliferative capacity and expression of markers related to antigen experience and dysfunction. This is an important caveat and finding that can partly explain why 3 of 6 PDX models *did not* respond to TIL therapy. It may also mean that TIL expansion protocols may need adaptions to make sure tumor-reactive TILs are enriched in cell product. The animal model described here is perfect to try out these methods because the same tumor can be treated with different cell products of the same patient biopsy. The model will also be very useful in future studies trying to genetically engineer the TILs to become better. This includes CRISPR knockout/knockin experiments targeting suppressive genes in TILs, chimeric antigen receptors, as well as cytokine and chemokine expression to enhance homing, survival, and proliferation. TILs do home to tumors and are therefore also optimal to use as delivery cells while being cytotoxic. The model described here is humanized, so any novel methodology developed can be translated to the clinic in an accelerated fashion.

## Supplementary Materials

Figures A1–A13

Table A1

Table A2

## References

[1] Bray F., Ferlay J., Soerjomataram I. (2018). Global cancer statistics 2018: GLOBOCAN estimates of incidence and mortality worldwide for 36 cancers in 185 countries. CA Cancer J Clin.

[2] Siegel R.L., Miller K.D., Jemal A. (2020). Cancer statistics, 2020. CA Cancer J Clin.

[3] McGuigan A., Kelly P., Turkington R.C. (2018). Pancreatic cancer: a review of clinical diagnosis, epidemiology, treatment and outcomes. World J Gastroenterol.

[4] Chen D.S., Irving B.A., Hodi F.S. (2012). Molecular pathways: next-generation immunotherapy--inhibiting programmed death-ligand 1 and programmed death-1. Clin Cancer Res.

[5] Keir M.E., Butte M.J., Freeman G.J. (2008). PD-1 and its ligands in tolerance and immunity. Annu Rev Immunol.

[6] Couzin-Frankel J. (2013). Breakthrough of the year 2013. Cancer immunotherapy. Science.

[7] Yang Y. (2015). Cancer immunotherapy: harnessing the immune system to battle cancer. J Clin Invest.

[8] Looi C.K., Chung F.F., Leong C.O. (2019). Therapeutic challenges and current immunomodulatory strategies in targeting the immunosuppressive pancreatic tumor microenvironment. J Exp Clin Cancer Res.

[9] Qin C., Yang G., Yang J. (2020). Metabolism of pancreatic cancer: paving the way to better anticancer strategies. Mol Cancer.

[10] Ren B., Cui M., Yang G. (2018). Tumor microenvironment participates in metastasis of pancreatic cancer. Mol Cancer.

[11] Feng M., Xiong G., Cao Z. (2017). PD-1/PD-L1 and immunotherapy for pancreatic cancer. Cancer Lett.

[12] Gong J., Hendifar A., Tuli R. (2018). Combination systemic therapies with immune checkpoint inhibitors in pancreatic cancer: overcoming resistance to single-agent checkpoint blockade. Clin Transl Med.

[13] Hidalgo M., Amant F., Biankin A.V. (2014). Patient-derived xenograft models: an emerging platform for translational cancer research. Cancer Discov.

[14] Gao H., Korn J.M., Ferretti S. (2015). High-throughput screening using patient-derived tumor xenografts to predict clinical trial drug response. Nat Med.

[15] Einarsdottir B.O., Bagge R.O., Bhadury J. (2014). Melanoma patient-derived xenografts accurately model the disease and develop fast enough to guide treatment decisions. Oncotarget.

[16] Gilles M.E., Hao L., Huang L. (2018). Personalized RNA medicine for pancreatic cancer. Clin Cancer Res.

[17] Izumchenko E., Paz K., Ciznadija D. (2017). Patient-derived xenografts effectively capture responses to oncology therapy in a heterogeneous cohort of patients with solid tumors. Ann Oncol.

[18] Patton E.E., Mueller K.L., Adams D.J. (2021). Melanoma models for the next generation of therapies. Cancer Cell.

[19] Ny L., Rizzo L.Y., Belgrano V. (2020). Supporting clinical decision making in advanced melanoma by preclinical testing in personalized immune-humanized xenograft mouse models. Ann Oncol.

[20] Rosenberg S.A., Yang J.C., Sherry R.M. (2011). Durable complete responses in heavily pretreated patients with metastatic melanoma using T-cell transfer immunotherapy. Clin Cancer Res.

[21] Hall M., Liu H., Malafa M. (2016). Expansion of tumor-infiltrating lymphocytes (TIL) from human pancreatic tumors. J Immunother Cancer.

[22] Jespersen H., Lindberg M.F., Donia M. (2017). Clinical responses to adoptive T-cell transfer can be modeled in an autologous immune-humanized mouse model. Nat Commun.

[23] Muralidharan S.V., Nilsson L.M., Lindberg M.F. (2020). Small molecule inhibitors and a kinase-dead expressing mouse model demonstrate that the kinase activity of Chk1 is essential for mouse embryos and cancer cells. Life Sci Alliance.

[24] Einarsdottir B.O., Karlsson J., Söderberg E.M.V. (2018). A patient-derived xenograft pre-clinical trial reveals treatment responses and a resistance mechanism to karonudib in metastatic melanoma. Cell Death Dis.

[25] Kim D., Langmead B., Salzberg S.L. (2015). HISAT: a fast spliced aligner with low memory requirements. Nat Methods.

[26] Li H., Handsaker B., Wysoker A. (2009). The sequence alignment/map format and SAMtools. Bioinformatics.

[27] Anders S., Pyl P.T., Huber W. (2015). HTSeq--a Python framework to work with high-throughput sequencing data. Bioinformatics.

[28] Bagge R.O., Demir A., Karlsson J. (2018). Mutational signature and transcriptomic classification analyses as the decisive diagnostic tools for a cancer of unknown primary. JCO Precis Oncol.

[29] Cancer Genome Atlas Research Network, Electronic address: andrew_aguirre@dfci.harvard.edu; Cancer Genome Atlas Research Network (2017). Integrated genomic characterization of pancreatic ductal adenocarcinoma. Cancer Cell.

[30] Racle J., de Jonge K., Baumgaertner P. (2017). Simultaneous enumeration of cancer and immune cell types from bulk tumor gene expression data. Elife.

[31] Li H., Durbin R. (2009). Fast and accurate short read alignment with Burrows-Wheeler transform. Bioinformatics.

[32] McKenna A., Hanna M., Banks E. (2010). The Genome Analysis Toolkit: a MapReduce framework for analyzing next-generation DNA sequencing data. Genome Res.

[33] Benjamin D., Sato T., Cibulskis K. (2019). Calling somatic SNVs and indels with Mutect2. bioRxiv.

[34] Karczewski K.J., Francioli L.C., Tiao G. (2020). The mutational constraint spectrum quantified from variation in 141,456 humans. Nature.

[35] Quintana E., Piskounova E., Shackleton M. (2012). Human melanoma metastasis in NSG mice correlates with clinical outcome in patients. Sci Transl Med.

[36] Waters A.M., Der C.J. (2018). KRAS: the critical driver and therapeutic target for pancreatic cancer. Cold Spring Harb Perspect Med.

[37] Muraro M.J., Dharmadhikari G., Grün D. (2016). A single-cell transcriptome atlas of the human pancreas. Cell Syst.

[38] Freeman G.J., Long A.J., Iwai Y. (2000). Engagement of the PD-1 immunoinhibitory receptor by a novel B7 family member leads to negative regulation of lymphocyte activation. J Exp Med.

[39] Sturm G., Finotello F., Petitprez F. (2019). Comprehensive evaluation of transcriptome-based cell-type quantification methods for immuno-oncology. Bioinformatics.

[40] Soares K.C., Rucki A.A., Wu A.A. (2015). PD-1/PD-L1 blockade together with vaccine therapy facilitates effector T-cell infiltration into pancreatic tumors. J Immunother.

[41] Lu C., Paschall A.V., Shi H. (2017). The MLL1-H3K4me3 axis-mediated PD-L1 expression and pancreatic cancer immune evasion. J Natl Cancer Inst.

[42] Zheng L. (2017). PD-L1 expression in pancreatic cancer. J Natl Cancer Inst.

[43] Yamaki S., Yanagimoto H., Tsuta K. (2017). PD-L1 expression in pancreatic ductal adenocarcinoma is a poor prognostic factor in patients with high CD8(+) tumor-infiltrating lymphocytes: highly sensitive detection using phosphor-integrated dot staining. Int J Clin Oncol.

[44] Lu C., Liu K. (2017). Epigenetic regulation of PD-L1 expression and pancreatic cancer response to checkpoint immunotherapy. Transl Cancer Res.

[45] Boyd C.A., Benarroch-Gampel J., Sheffield K.M. (2012). 415 Patients with adenosquamous carcinoma of the pancreas: a population-based analysis of prognosis and survival. J Surg Res.

[46] Hester C.A., Augustine M.M., Choti M.A. (2018). Comparative outcomes of adenosquamous carcinoma of the pancreas: an analysis of the National Cancer Database. J Surg Oncol.

[47] Katz M.H., Taylor T.H., Al-Refaie W.B. (2011). Adenosquamous versus adenocarcinoma of the pancreas: a population-based outcomes analysis. J Gastrointest Surg.

[48] Silvestris N., Brunetti O., Pinto R. (2018). Immunological mutational signature in adenosquamous cancer of pancreas: an exploratory study of potentially therapeutic targets. Expert Opin Ther Targets.

[49] Steele N.G., Biffi G., Kemp S.B. (2021). Inhibition of Hedgehog signaling alters fibroblast composition in pancreatic cancer. Clin Cancer Res.

[50] Shevchenko I., Mathes A., Groth C. (2020). Enhanced expression of CD39 and CD73 on T cells in the regulation of anti-tumor immune responses. Oncoimmunology.

[51] Seifert A.M., Eymer A., Heiduk M. (2020). PD-1 expression by lymph node and intratumoral regulatory T cells is associated with lymph node metastasis in pancreatic cancer. Cancers (Basel).

[52] Steele N.G., Carpenter E.S., Kemp S.B. (2020). Multimodal mapping of the tumor and peripheral blood immune landscape in human pancreatic cancer. Nat Cancer.

[53] Sivakumar S., Abu-Shah E., Ahern D.J. (2021). Activated regulatory T-cells, dysfunctional and senescent T-cells hinder the immunity in pancreatic cancer. Cancers (Basel).

[54] Poschke I.C., Hassel J.C., Rodriguez-Ehrenfried A. (2020). The outcome of ex vivo TIL expansion is highly influenced by spatial heterogeneity of the tumor T-cell repertoire and differences in intrinsic in vitro growth capacity between T-cell clones. Clin Cancer Res.

