# Supplementary material for: Genetics and Therapeutic Responses to Tumor-Infiltrating Lymphocyte Therapy of Pancreatic Cancer Patient-Derived Xenograft Models
Source: Gastro Hep Adv. 2022 Jul 15;1(6):1037–48. doi: 10.1016/j.gastha.2022.07.006 (PMC11307969; doi:10.1016/j.gastha.2022.07.006)
Supplement: Figures A1–A13 [file mmc1.pdf]

Supplemental information to Nilsson/Vilhav et al.

Figures

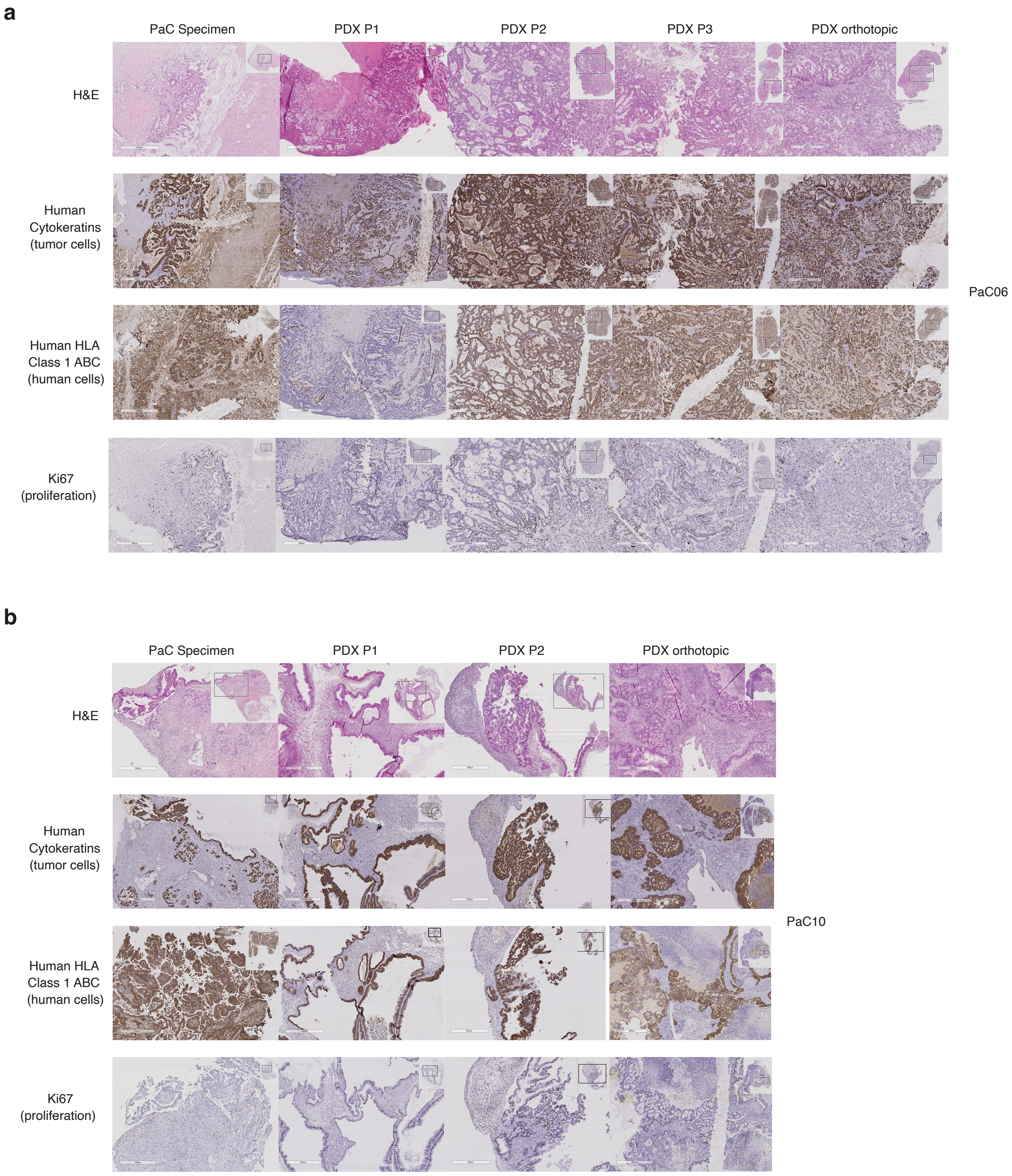

Supplemental Figure S1. IHC of pancreatic cancer in PaC06 and PaC10 (patient 6 and 10) and in after 2-3 passages in mice growing subcutaneously or orthotopically.

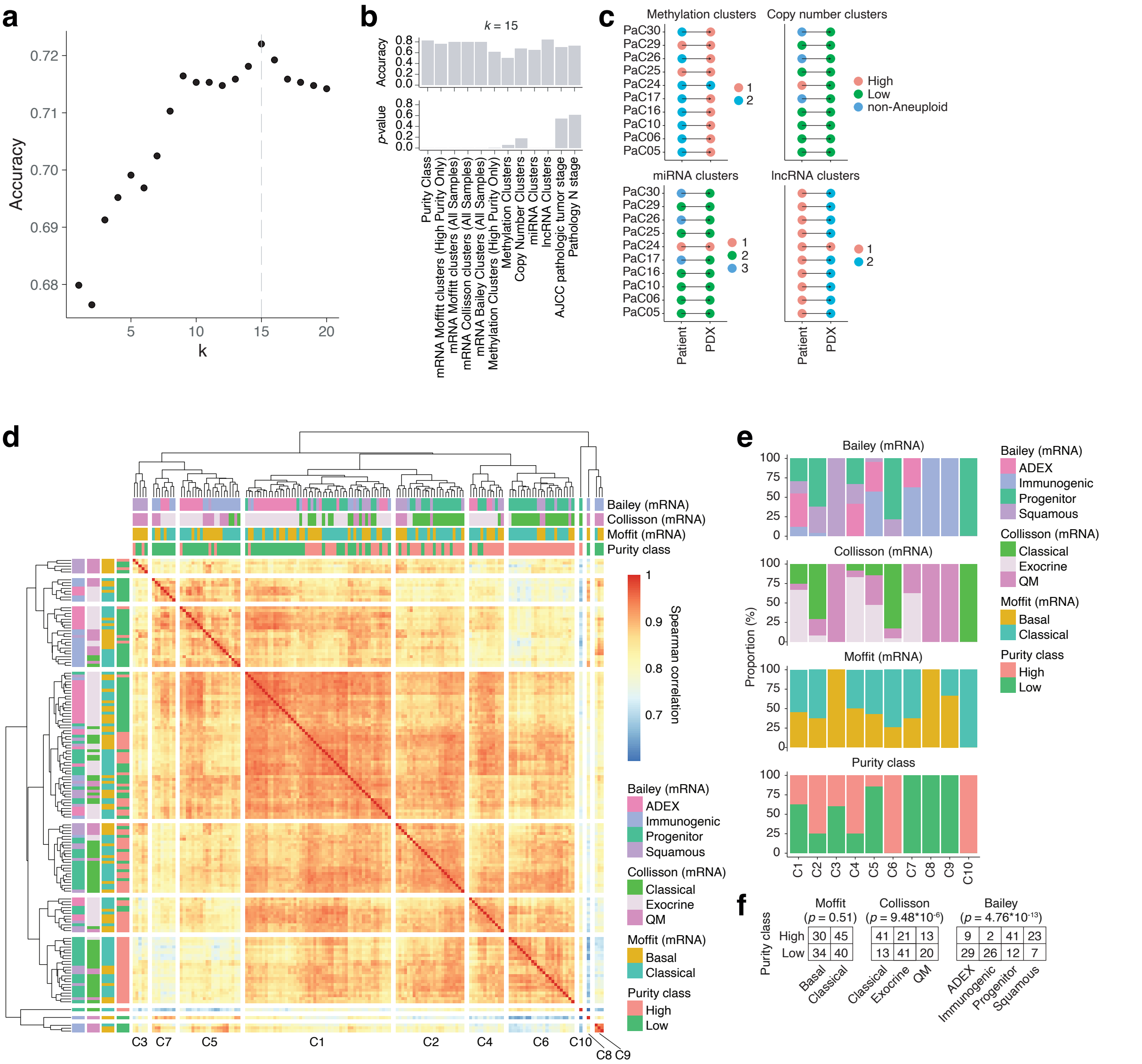

Supplemental Figure S2. Classification of pancreatic cancer subtypes. **a)** Leave-one-out cross-validation on the TCGA-PAAD cohort to find an optimal value of the parameter k, for k-NN analysis. $k = 15$ was selected based on the highest average accuracy across all categories used for prediction. **b)** Accurary and p-values (accuracy relative to no information rate, calculated by the *confusionMatrix* function in the *caret* R package [54]) indicating the performance per category, as described in (a). **c)** Classification of subtypes for our samples, as in **Fig. 2e**, showing additional categories assessed. **d**) Clustering of samples from the TCGA PAAD cohort based on genes identified as differentially expressed between biopsy and PDX with DESeq2 at $q < 0.05$. Samples are annotated based on pre-defined purity classes and subtypes. e) Relative representation of different subtypes and purity classes in each cluster from (d). f) Fisher tests with respect to associations between subtype classification schemes and sample purity.

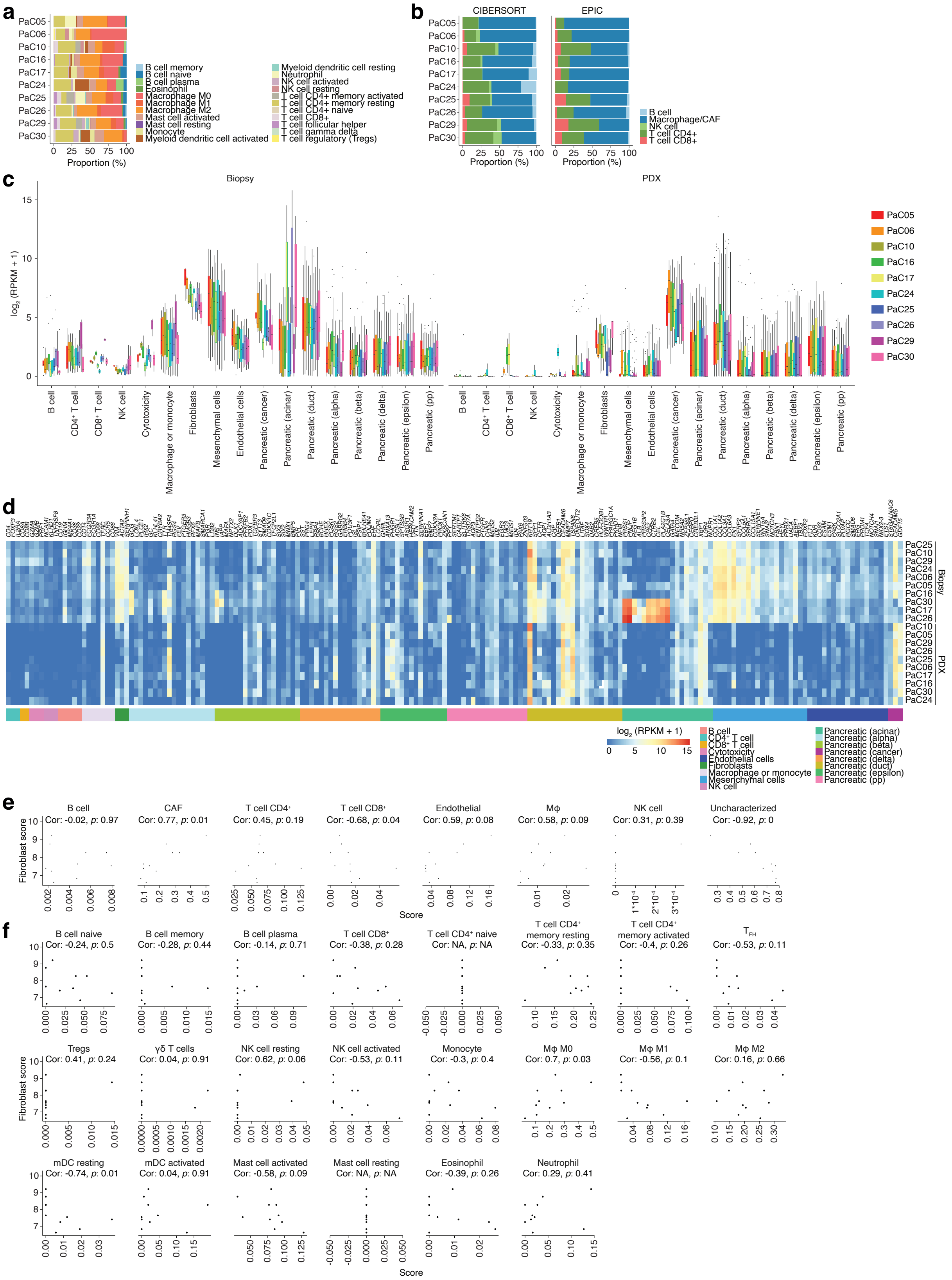

Supplemental Figure S3: Comparison between EPIC and CIBERSORT for cell type deconvolution. a) Cell type proportions relative to the total immune cell content inferred by CIBERSORT. b) Proportions of cell types that are both predicted by CIBERSORT and EPIC, after merging the categories macrophages and cancer-associated fibroblasts (CAF). c) Expression of markers of different cell types in the tumour microenvironment of biopsies and corresponding levels of these in PDX samples. Markers of pancreatic cell subsets and

mesenchymal cells were selected from public available single-cell data of the pancreas. d) Detailed expression of each of the markers in (c), annotated with their respective cell types. e) Correlation of the average expression of the fibroblast markers in (c-d, "Fibroblast score") against inferred cell type proportions with EPIC and f) CIBERSORT. Correlations coefficients are calculated using Spearman correlation.

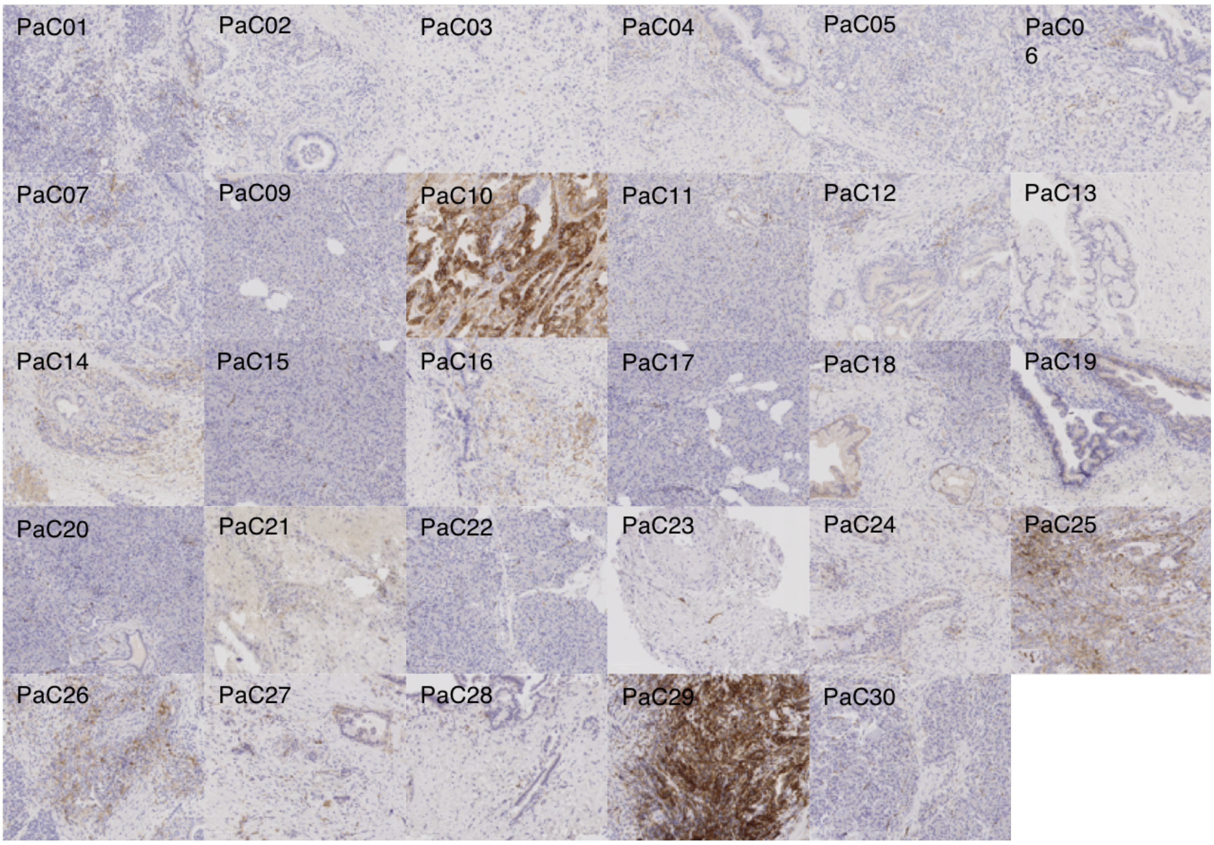

Supplemental Figure S4. Immunohistochemistry stainings for PD-L1 expression in pancreatic cancer biopsies of all patient in the study.

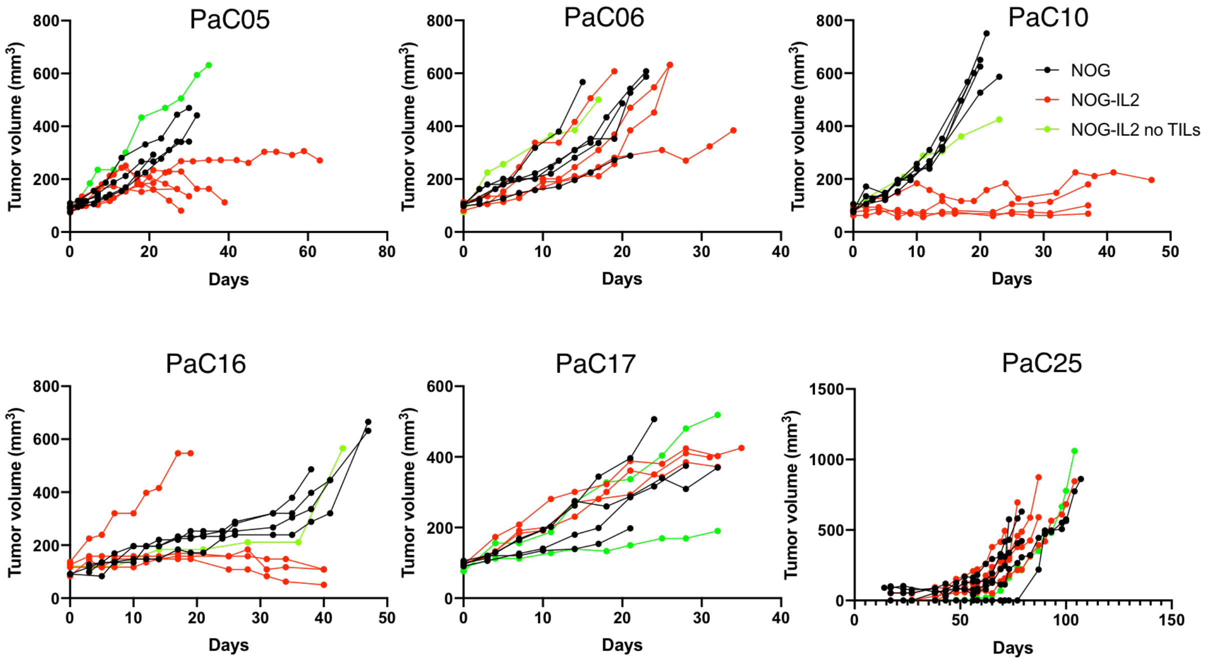

Supplemental Figure S5. Tumor growth curves of pancreatic cancer PDX tumors (n=3-5) growing in NOG mice or in hIL2-NOG mice and treated with autologous TILs. Shown are individual growth curves including controls where tumors are grown in NOG or hIL2-NOG without TILs. Cumulative growth curves are shown in **Fig. 4**.

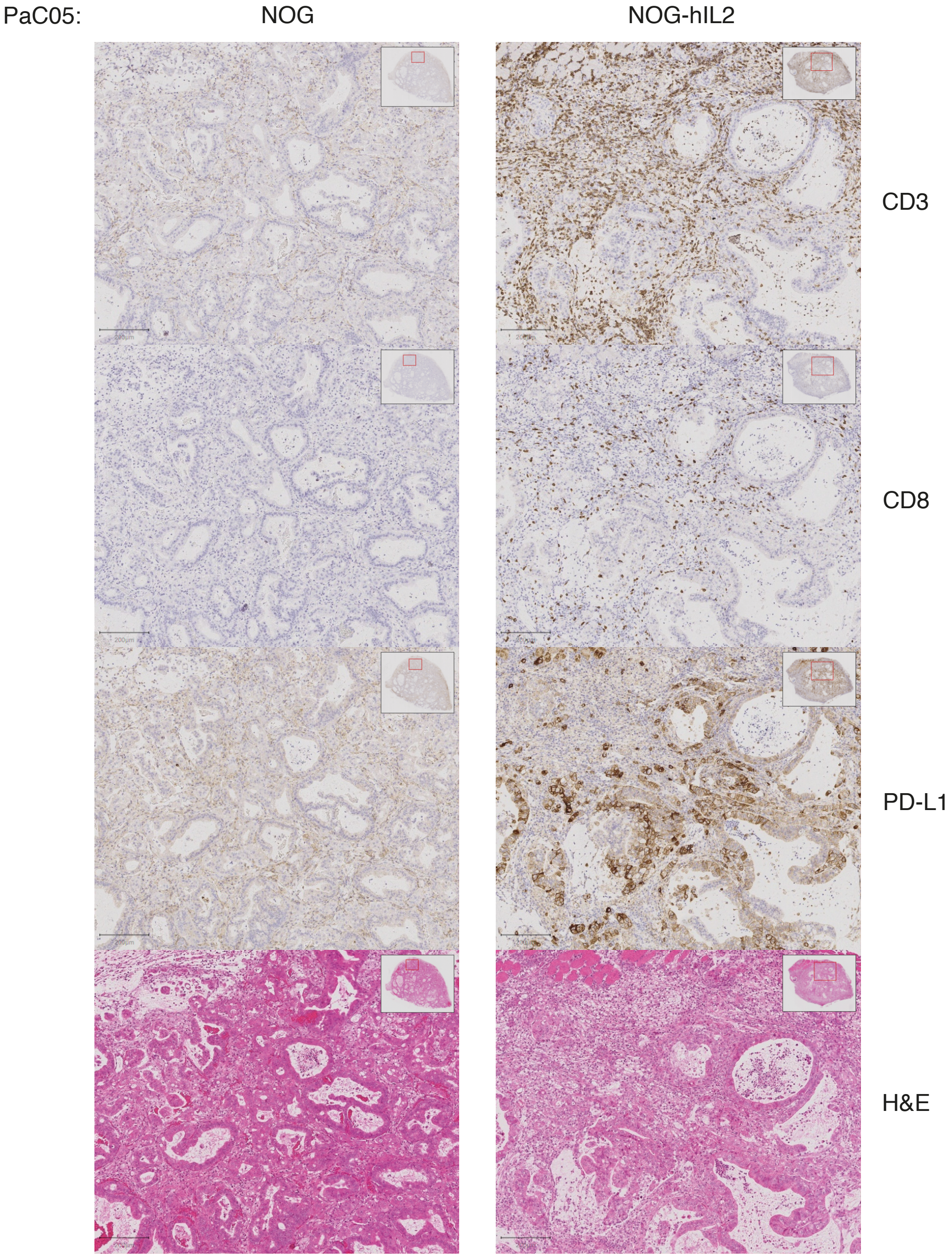

Supplemental Figure S6. Immunohistochemistry stainings for CD3, CD8, PD-L1 and H&E of one NOG and one hIL2-NOG mouse of PaC05. TILs were only given to the hIL2-NOG mouse.

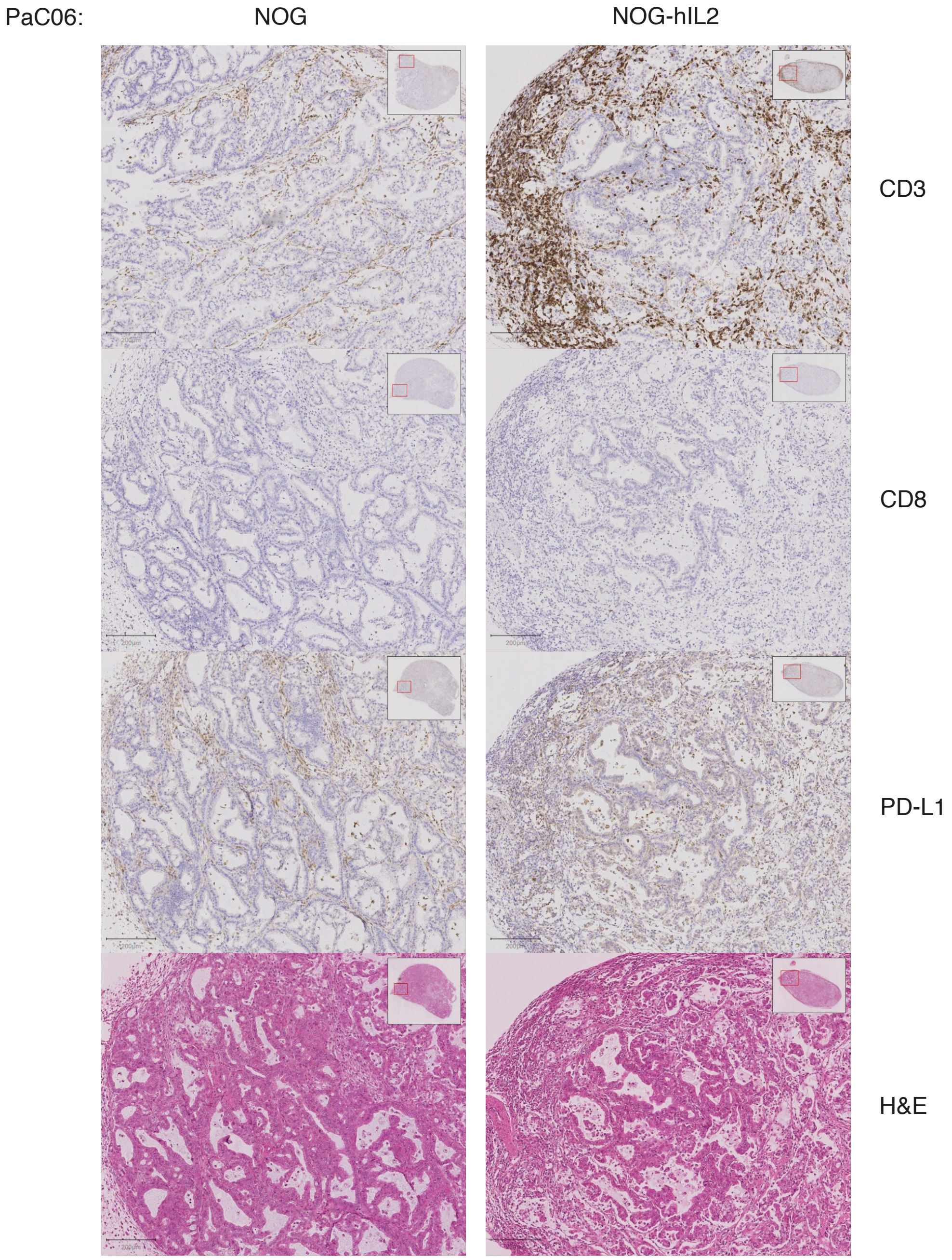

Supplemental Figure S7. Immunohistochemistry stainings for CD3, CD8, PD-L1 and H&E of one NOG and one hIL2-NOG mouse of PaC06. TILs were only given to the hIL2-NOG mouse.

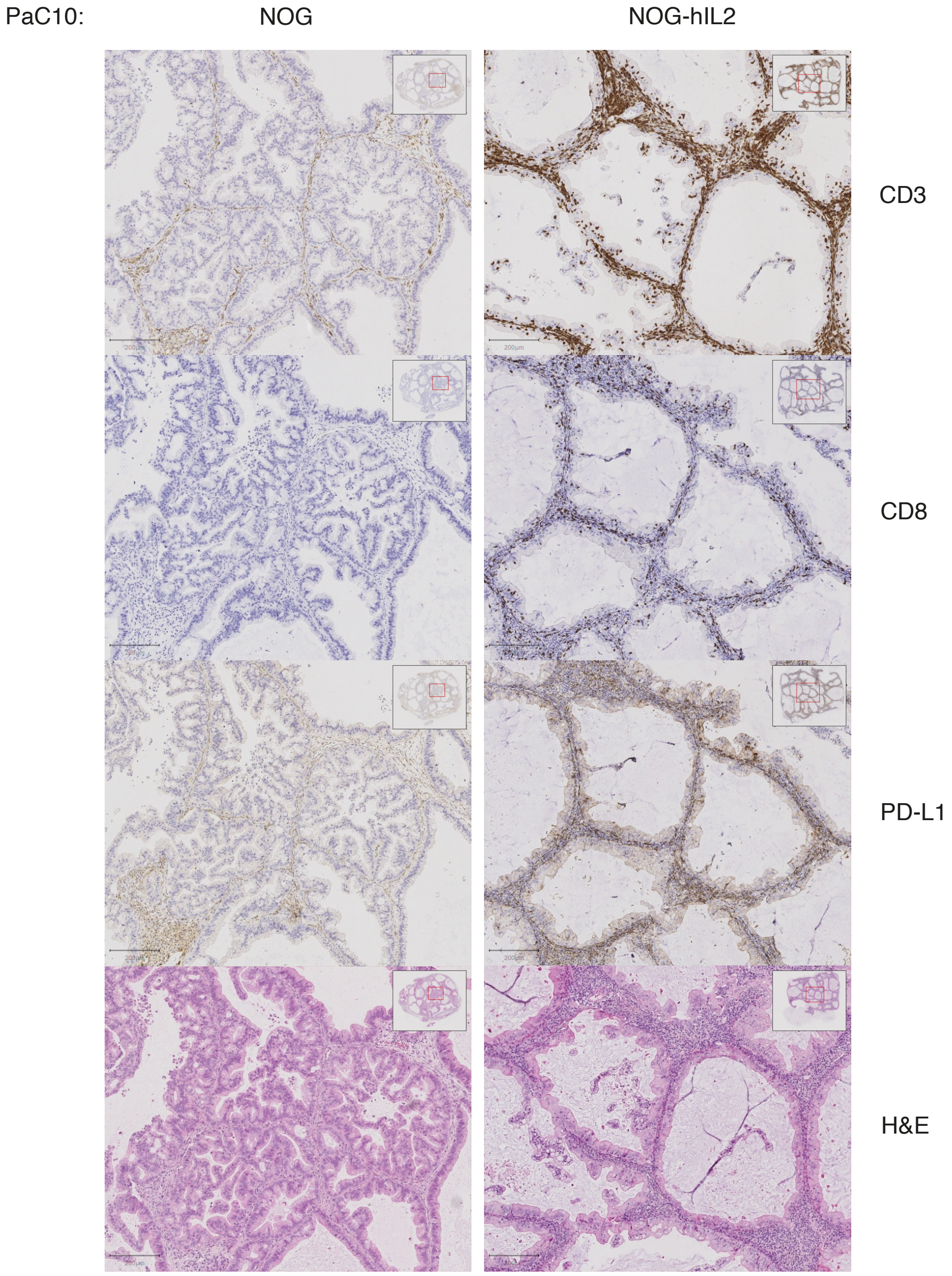

Supplemental Figure S8. Immunohistochemistry stainings for CD3, CD8, PD-L1 and H&E of one NOG and one hIL2-NOG mouse of PaC10. TILs were only given to the hIL2-NOG mouse.

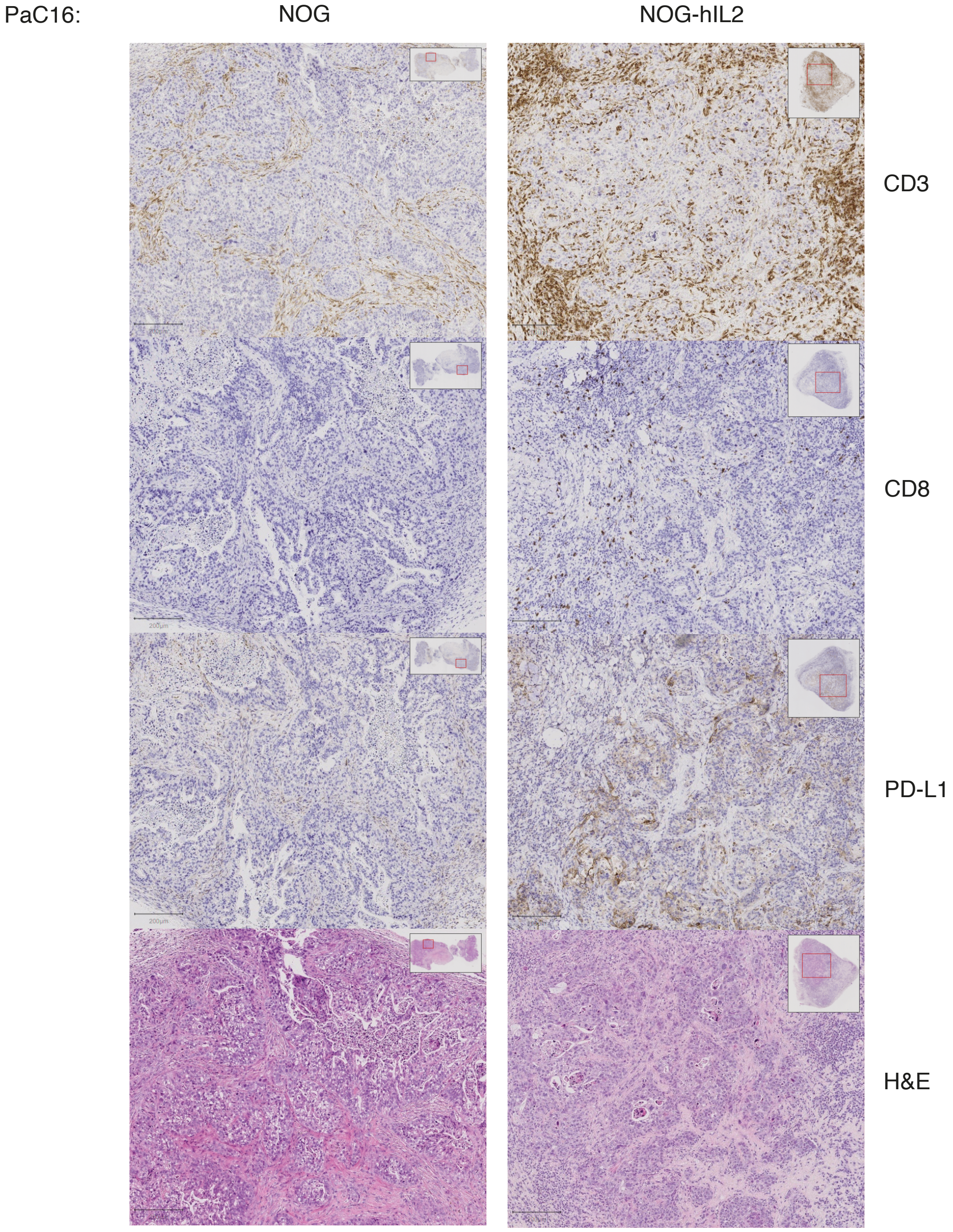

Supplemental Figure S9. Immunohistochemistry stainings for CD3, CD8, PD-L1 and H&E of one NOG and one hIL2-NOG mouse of PaC16. TILs were only given to the hIL2-NOG mouse.

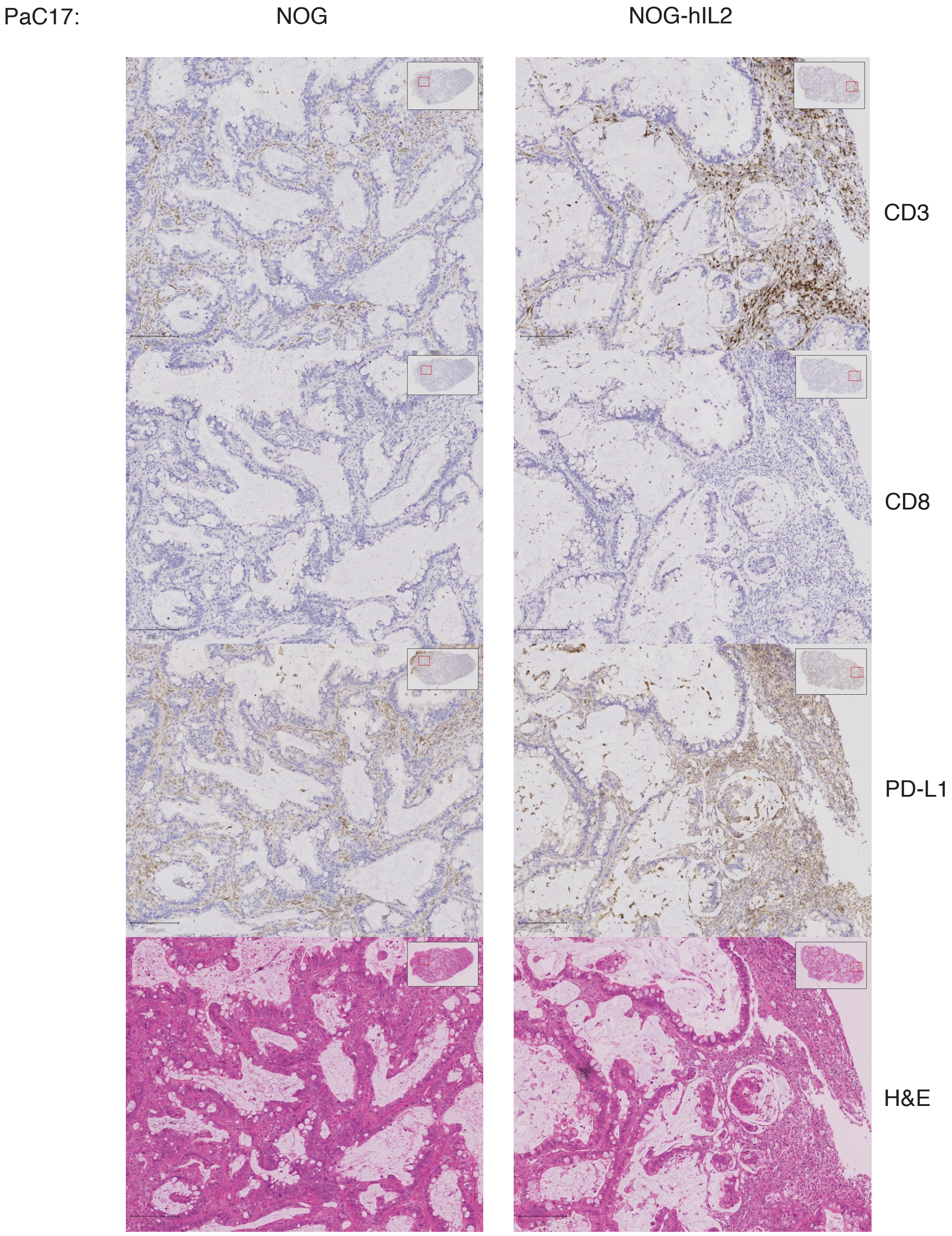

Supplemental Figure S10. Immunohistochemistry stainings for CD3, CD8, PD-L1 and H&E of one NOG and one hIL2-NOG mouse carrying subcutaneously growing PaC17. TILs were only given to the hIL2-NOG mouse.

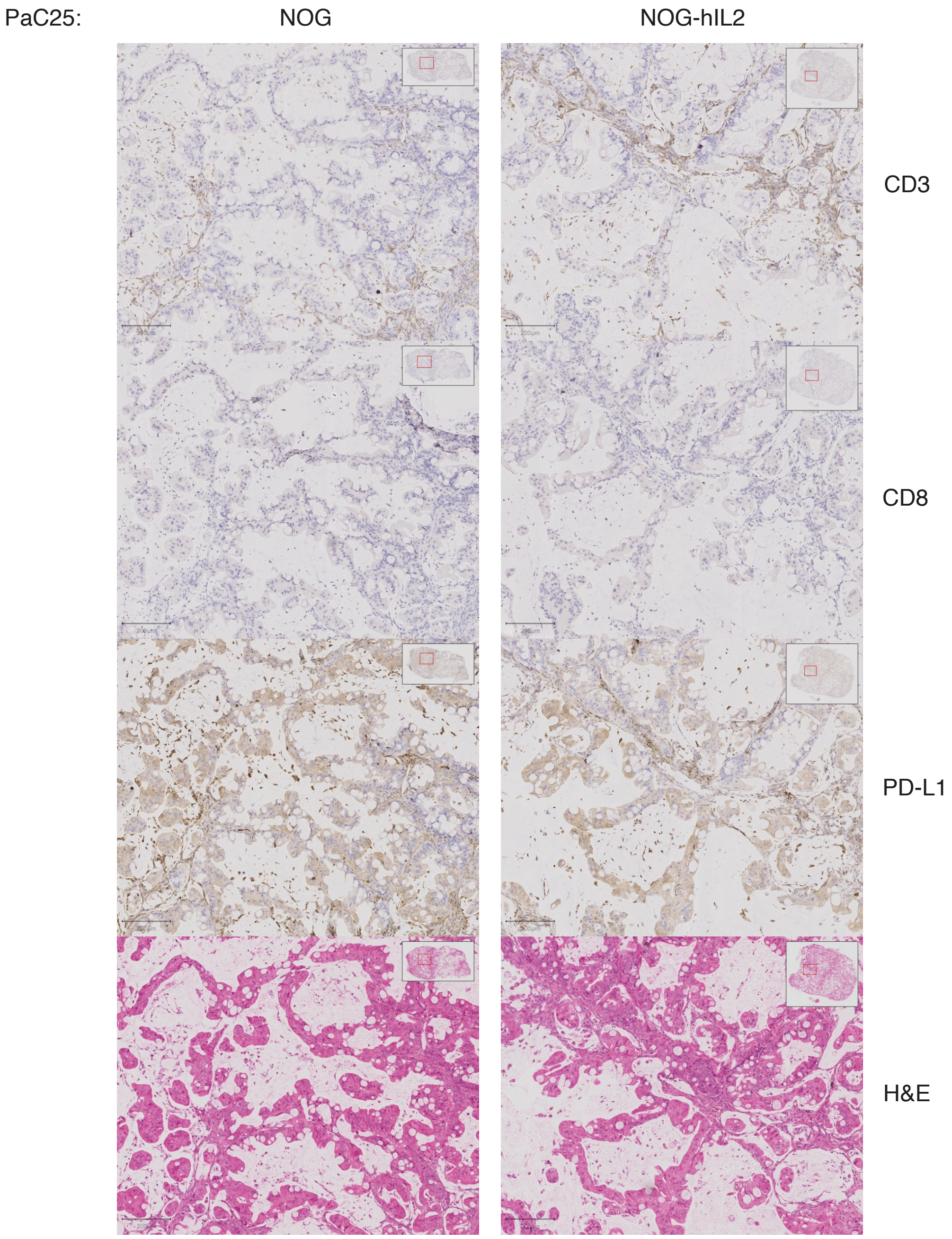

Supplemental Figure S11. Immunohistochemistry stainings for CD3, CD8, PD-L1 and H&E of one NOG and one hIL2-NOG mouse carrying subcutaneously growing PaC25. TILs were only given to the hIL2-NOG mouse.

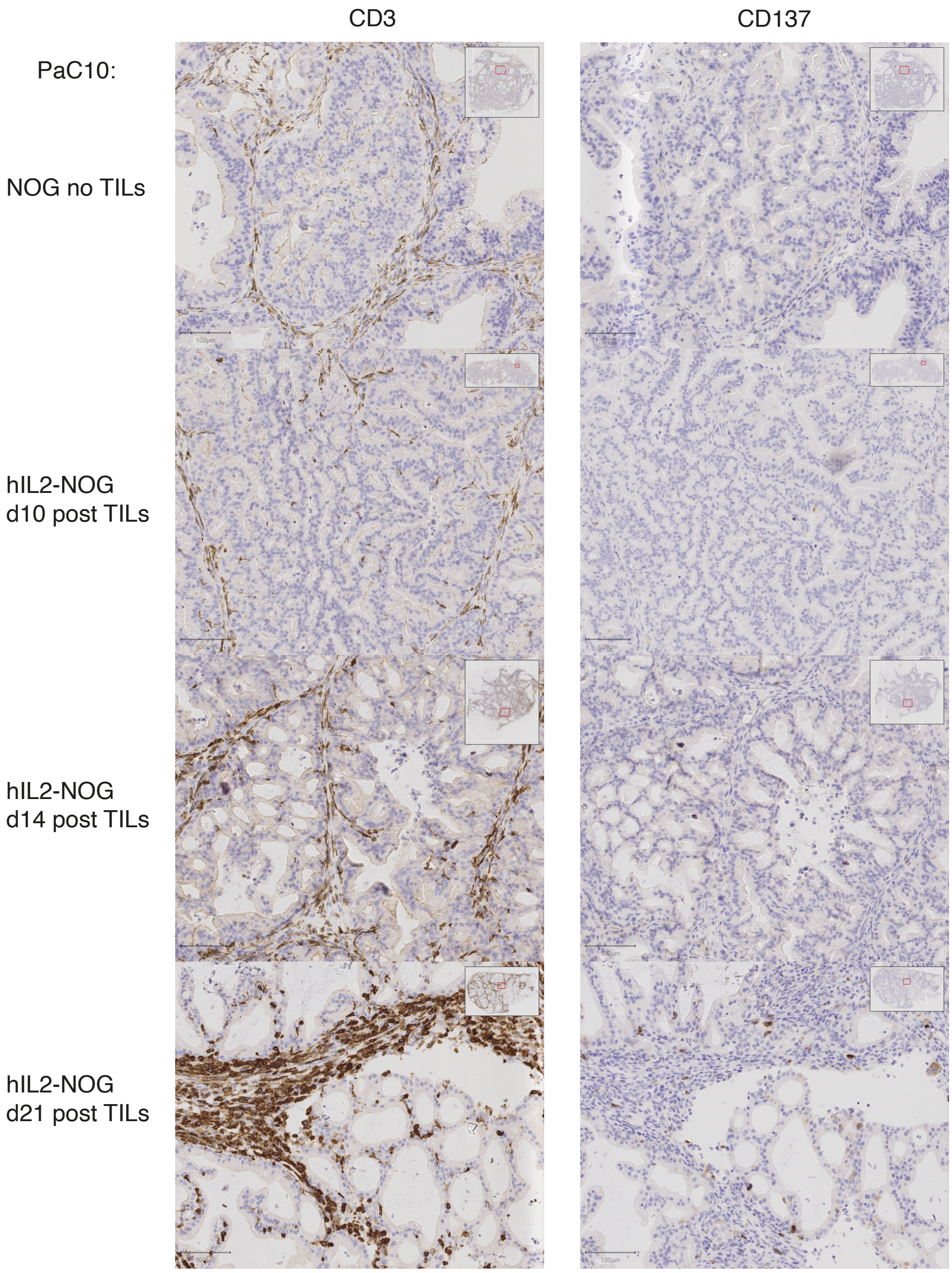

Supplemental Figure S12. Immunohistochemistry stainings showing expression of CD3 and CD137 (4-1BB) in mice carrying PaC10 and autologous TILs. One NOG mouse did not get any TILs and the hIL2-NOG mice all received $10x10^6$ TILs and were harvested at indicated time points after treatment.

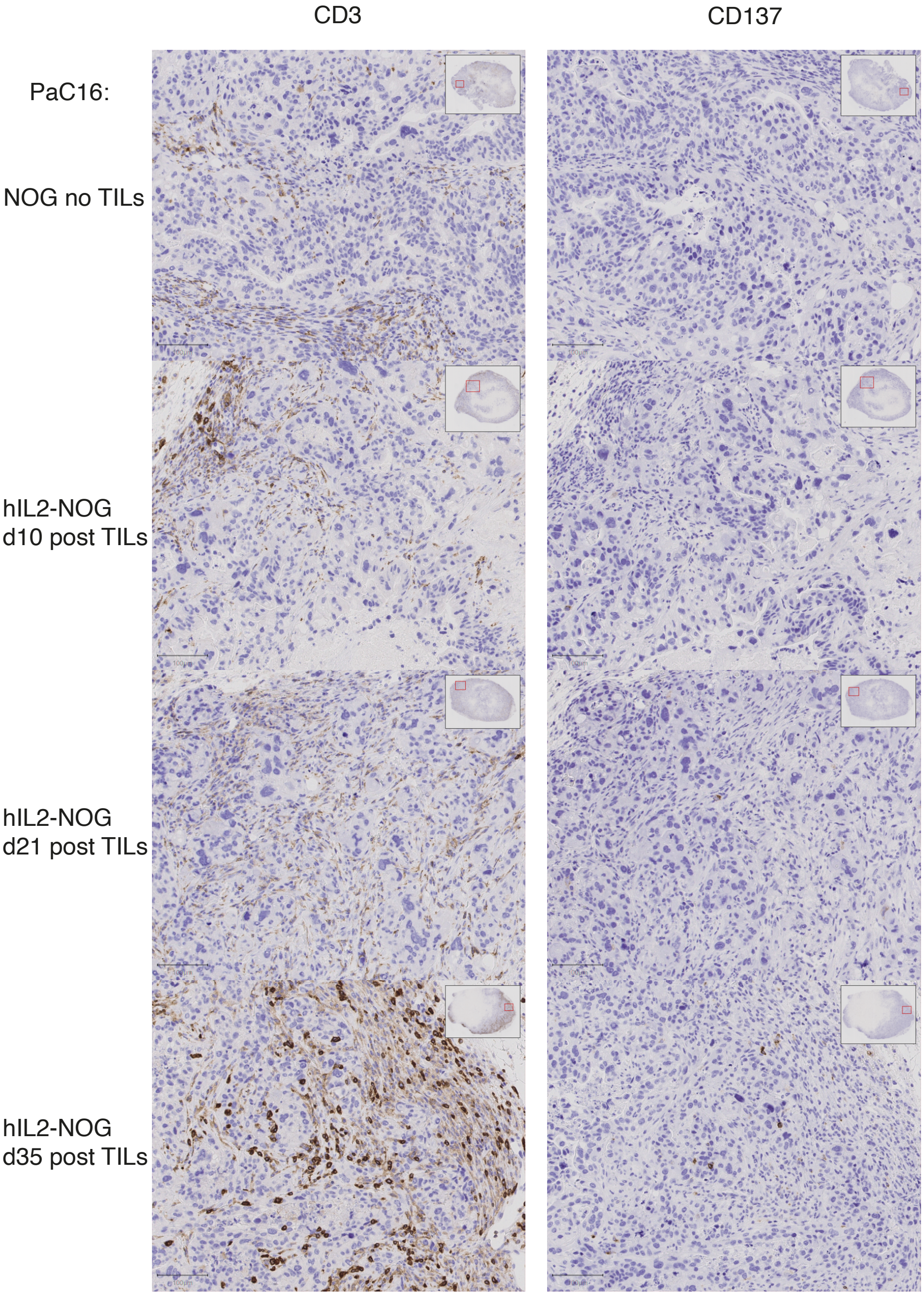

Supplemental Figure S13. Immunohistochemistry stainings showing expression of CD3 and CD137 (4-1BB) in mice carrying PaC16 and autologous TILs. One NOG mouse did not get any TILs and the hIL2-NOG mice all received 10x10^6 TILs and were harvested at indicated time points after treatment.
